# Current Status of Metabolic Compliance and Risk of Cardiovascular Disease in Patients with Type 2 Diabetes in the Zhuang Population in China

**DOI:** 10.1155/2023/1057121

**Published:** 2023-12-22

**Authors:** Danqing Xu, Xia Dai, Qiong Yang, Xueying Li, Ying Xiao, Qiuhong Huang, Qingqing Lou

**Affiliations:** ^1^The First Affiliated Hospital of Guangxi Medical University, Nanning 530021, China; ^2^The Second Affiliated Hospital of Guangxi Medical University, Nanning 530005, China; ^3^The First Affiliated Hospital of Hainan Medical University, Haikou 570101, China

## Abstract

**Introduction:**

This study aimed to investigate the blood glucose, blood pressure, and blood lipid status in Zhuang patients with T2DM and to analyze the correlation between compliance with metabolic monitoring and cardiovascular risk factors.

**Methods:**

A total of 1975 Zhuang patients with T2DM were evaluated in four Class III Grade A hospitals in three prefecture-level cities in the Guangxi Zhuang Autonomous Region between January and August 2022. Laboratory indicators, lifestyle, and demographic characteristics were collected.

**Results:**

The compliance rates for blood glucose, blood pressure, and blood lipids were 26.08%, 45.77%, and 30.58%, respectively, and only 5.06% of the patients reached the standard in all three indices. The compliance rates for blood glucose, blood pressure, and blood lipids in the CVD group were 32.92%, 21.74%, and 9.94%, respectively. In the CVD group, the usage rates of hypoglycemic, antihypertensive, and lipid-lowering drugs were 77.54%, 3.17%, and 4.11%, respectively. Binary logistic regression analysis showed that older age (OR = 1.033, 95% CI [1.016, 1.050]), female (OR = 0.402, 95% CI [0.260, 0.621]), smoke (OR = 1.994, 95% CI [1.361, 2.922]), blood pressure noncompliance + use of antihypertensive drugs (OR = 0.348, 95% CI [0.230, 0.527]), and blood lipid noncompliance + use of lipid-lowering drugs (OR = 0.244, 95% CI [0.142, 0.417]) were risk factors for CVDs, and moderate-intensity exercise (OR = 0.439, 95% CI [0.300,0.640]) was protective against CVD.

**Conclusions:**

Older age, female, smoke, blood lipid levels, and blood pressure noncompliance were risk factors for CVD while moderate-intensity exercise was observed to be protective.

## 1. Introduction

China is ranked first globally in terms of diabetic patients with 141 million cases in 2021, according to data from the International Diabetes Federation (IDF) [1]. Diabetes carries a significant risk of cardiovascular disease (CVD), and cardiovascular complications are now the primary cause of mortality in diabetic patients. In China, the Zhuang population in the Guangxi Zhuang Autonomous Region differs from the predominant Han population in terms of diabetes-related genotypes [2], demographic characteristics, economic level, dietary habits, and educational level. Most Zhuang people live in mountainous areas where transportation is difficult. Their main grain crops are rice and corn, and they like to drink alcohol and plant tobacco. Studies have reported that a high intake of dietary fiber can reduce the risk of CVD, T2DM, and some cancers [3]. However, smoking, drinking, and other unhealthy habits are associated with an increased incidence of hypertension, dyslipidemia, diabetes, and obesity, which may further increase the morbidity and mortality from CVD in China [4]. According to previous studies, the Zhuang population has high incidences of T2DM, hyperlipidemia, hypertension, and other diseases [5]. Controlling blood pressure and blood glucose and lipid levels can significantly reduce the incidence of CVD. Diabetes, complicated by cardiovascular and cerebrovascular diseases, has amplified the medical burden on society. It is thus important to control the risk factors of diabetes and CVD. This study investigated the risk factors for CVD in Zhuang T2DM patients. The associations between the status of blood pressure, blood glucose and lipid standards, and cardiovascular risk factors were evaluated in Zhuang T2DM patients in Guangxi to provide a theoretical basis and guidance for the metabolic control and prevention of CVD in this region.

## 2. Subjects and Methods

### 2.1. Subjects

A retrospective analysis was performed on a total of 1975 Zhuang patients with T2DM from four Class III Grade A hospitals in three prefecture-level cities in the Guangxi Zhuang Autonomous Region in 2022. The inclusion criteria were as follows: (1) the patients were of Zhuang ethnicity, shown by the direct third generation belonging to Zhuang ethnicity; (2) the patients were aged between 22 and 87 years; (3) the patients had been diagnosed with T2DM for ≥3 months before data collection; and (4) the patients had lived in Guangxi for over five years. The exclusion criteria were as follows: (1) pregnant and nursing women and (2) patients with mental illness and communication difficulties. All patients voluntarily participated in the investigation and signed written informed consent.

### 2.2. Methods

General information and biochemical indicators such as glycated hemoglobin or hemoglobin A1c (HbA1c), blood pressure, low-density lipoprotein cholesterol (LDL-C), high-density lipoprotein cholesterol (HDL-C), and total cholesterol (TC), as well as drinking history, family history, smoking history, and disease history, were collected from the electronic medical records. A telephone interview was conducted if there were missing values or incorrect data. Two nursing professionals were chosen from each hospital to collect data. Two individuals were responsible for data entry, and 10% of patients' data were randomly selected for verification.

### 2.3. Definitions and Diagnostic Criteria

T2DM was diagnosed according to the 2022 diagnostic criteria of the American Diabetes Association (ADA) [6]. The 10-year risk of ischemic CVD (ICVD) was calculated using a 10-year risk assessment table recommended by the Chinese Guidelines for the Prevention of Cardiovascular Diseases, which included information on age, systolic blood pressure (SBP), TC, body mass index (BMI), diabetes, and smoking history, leading to a determination of the absolute risk level of ICVD within 20 years, with an absolute risk score <5% representing low risk, ≥5% and <10% indicating medium risk, and ≥10% high risk [7]. Smokers were defined as those who smoked at least one cigarette per day, and those who had not smoked within the last year were counted as nonsmokers. Alcohol consumption was defined as drinking more than 30 g of alcohol per day and drinking for more than one year. Cycling, jogging, playing sports, skiing, swimming, and other activities with a metabolic equivalent (MET) expenditure of >3.0 were considered moderate-intensity exercise. The following criteria were used to diagnose CVD [8]: congestive heart failure; stroke; and coronary artery disease (CAD), including typical angina pectoris and myocardial infarction. Blood lipids, blood pressure, and blood sugar compliance standards were in accordance with the ADA's 2021 diagnostic criteria [9]. The blood glucose criteria included HbA1c < 7%, the blood pressure criteria were BP < 140/90 mmHg, and the blood lipid criteria were LDL-C < 2.6 mmol/L in the absence of a clear diagnosis of CVD, while blood glucose of HbA1c < 7%, blood pressure of BP < 130/80 mmHg, and blood lipid levels of LDL-C < 1.8 mmol/L were used when CVD was diagnosed. Abnormal blood glucose was defined as HbA1c < 7% and in those taking hypoglycemic drugs as HbA1c ≥ 7%. The definitions of abnormal blood pressure were as follows: with a clear diagnosis of CVD, BP ≥ 130/80 mmHg or BP < 130/80 mmHg with the use of antihypertensive drugs; in the absence of a clear CVD diagnosis, BP ≥ 140/90 mmHg or BP < 140/90 mmHg when taking antihypertensive drugs. The definitions of dyslipidemia are as follows: with a clear diagnosis of CVD, LDL-C ≥ 1.8 mmol/L or LDL-C < 1.8 mmol/L when using lipid-lowering drugs; in the absence of a clear CVD diagnosis, LDL-C ≥ 2.6 mmol/L or LDL-C < 2.6 mmol/L when using lipid-lowering drugs.

### 2.4. Statistical Methods

SPSS 27.0 was used for data sorting and analysis. Enumeration data were expressed as a constituent ratio (%), and comparisons between and within groups were performed by *χ*^2^ tests; measurement data were expressed as mean ± standard deviation and compared by independent sample *t*-tests. A binary logistic regression model using CVD as the dependent variable and the non-CVD patient group serving as the control group was used to evaluate the factors influencing CVD. Sex, age, smoke ICVD 10-year absolute risk, family history of diabetes, moderate-intensity exercise, blood pressure compliance, and blood lipid compliance were taken as independent variables. The forward selection method was used to select independent variables. The probability of including a variable in the model was *α* ≤ 0.05, and that for excluding was *α* > 0.1. *P* < 0.05 was considered statistically significant.

## 3. Results

### 3.1. General Information about the Subjects

A total of 2013 Zhuang patients with T2DM were initially included in the study. Of these, 38 patients with incomplete data were excluded, resulting in the inclusion of 1975 patients in the final analysis. The average age was 58.89 ± 11.24 years, the average duration of diabetes was 6.01 ± 4.93 years, and the average age at diagnosis of diabetes was 53.99 ± 11.38 years. There were 1328 males (67.24) in total. The CVD group contained 161 cases (8.15%), and the non-CVD group included 1814 cases (91.85%). Among the patients with CVD events, 131 patients (81.37%) had had a stroke, 15 patients (9.32%) had experienced an acute myocardial infarction, and 15 patients (9.32%) had coronary heart disease. There were significant differences in age, sex, SBP, the 10-year absolute risk of ICVD, family history of diabetes, moderate-intensity exercise, smoking, blood pressure, and blood lipids between the two groups (*P*  <  0.05). Among the 1975 patients, 1136 (57.52%) had moderate or above 10-year absolute risk of ICVD, of which 125 patients (77.64%) were in the CVD group and 1011 (55.74%) were in the non-CVD group, The compliance rates of blood pressure (21.74% vs. 47.91%) and LDL-C (9.94% vs. 32.41%) in the CVD group were lower than those in the non-CVD group, Among the 1975 patients, the compliance rates of blood glucose, blood pressure, and blood lipid were 26.08%, 45.77%, and 30.58%, respectively. Only 100 patients (5.06%) complied with all three items of blood pressure, blood glucose, and lipids, as shown in Table 1.

### 3.2. Status of Abnormal Blood Pressure, Blood Lipids, and Blood Glucose

The compliance rates of blood glucose, blood pressure, and blood lipids in the CVD group were 14.29%, 21.74%, and 9.32%, respectively. The rates of usage of hypoglycemic, antihypertensive, and lipid-lowering drugs were 77.54%, 3.17%, and 4.11%, respectively. The usage rates of hypoglycemic drugs (77.54% vs. 79.47%) in blood glucose noncompliant cases, antihypertensive drugs (3.17% vs. 4.74%) in blood pressure non-compliant cases, and lipid-lowering drugs (4.11% vs. 4.74%) in blood lipids noncompliant cases in the CVD group were lower than those in the non-CVD group, and the usage of angiotensin-converting enzyme inhibitors (ACEIs)/angiotensin receptor blockers (ARBs) was also lower than that in the non-CVD group (0.08% vs. 0.32%). As shown in Table 2, less than 10% of the patients took lipid-lowering medications, and statins were the medication of choice for all 65 patients who did take lipid-lowering medications.

### 3.3. Binary Logistic Regression Analysis of CVD Risk Factors

According to the results, age (OR = 1.033, 95% CI [1.016, 1.050]), gender (OR = 0.402, 95% CI [0.260, 0.621]), moderate-intensity exercise (OR = 0.439, 95% CI [0.300, 0.640]), smoke (OR = 1.994, 95% CI [1.361, 2.922]), use of antihypertensive drugs + blood pressure noncompliance (OR = 0.348, 95% CI [0.230, 0.527]), and use of lipid-lowering drugs + blood lipids non-compliance (OR = 0.244, 95% CI [0.142, 0.417]) were associated with CVD, as shown in Table 3.

## 4. Discussion

In daily life, diabetes and cardiovascular diseases not only significantly affect patients' quality of life but also bring a severe burden to society and families. Poor metabolic control increases the risk of diabetic complications, while reasonable metabolic control helps stabilize the patient's condition. The results of this study showed that the blood glucose, blood pressure, and blood lipids compliance rates of Zhuang patients were 26.08%, 45.77%, and 30.58%, respectively. The compliance rates of blood lipids and blood glucose from our study are higher than those in Tibetan T2DM patients in Yan Mao's survey, which reported the compliance rates of blood lipids and blood glucose at 8.0% and 11.0%, respectively [10]. The reason may be the short Course of diabetes mellitus used (6 years vs 10 years) and the inclusion of moderate-intensity exercise. It may also be due to the higher educational level of the patients. The blood glucose compliance rate was lower than that of Chinese outpatients with T2DM (32.6%) [11], and the blood pressure and blood lipid compliance rates were much lower than the results of community T2DM patients (compliance rates of blood pressure and blood lipids were of 31.4% and 39.4%, respectively) in Nanjing in Liu Jie's survey [12]. Poor dietary adherence, a lack of self-management, the state of the economy, and failure to take medication may all be factors influencing compliance rates. Only 5.06% of Guangxi Zhuang T2DM patients met the standards for blood pressure, blood lipids, and glucose simultaneously, suggesting that the level of comprehensive management needs to be raised. These results are lower than those reported in Liu Lin's survey on Chinese patients with T2DM in 2010 (5.4%) [13]. Possible explanations include the location of the selected research site in Guangxi, western China, where the economic level is relatively low and knowledge of diabetes prevention and treatment is relatively poor. Therefore, patients will typically present at hospitals only when the disease has progressed considerably. Despite significant improvements over the past decade, the overall control rate of blood glucose, blood pressure, and blood lipids in patients with diabetes in the Zhuang population remains unsatisfactory. Hence, there is substantial room for further improvement in diabetic metabolic control to reduce the morbidity and mortality of diabetes-related CVDs.

The results of this study showed that the compliance rates of blood glucose, blood pressure, and blood lipids in the CVD group were 32.92%, 21.74%, and 9.94%, respectively. The usage rates of hypoglycemic, antihypertensive, and lipid-lowering drugs were 77.54%, 3.17%%, and 4.11%%, respectively. Studies have indicated that normal high blood pressure can increase the risk of coronary atherosclerotic heart disease, myocardial infarction, stroke and other diseases [14]. Diabetes mellitus patients can control blood glucose-related indicators within the normal range to prevent or delay the occurrence and development of complications through effective management and nursing intervention, such as medication, dietary management, blood glucose monitoring, physical activity, and treatment of complications. This study shows that the current blood glucose control and the clinical medication situation in Zhuang T2DM patients are unsatisfactory. Therefore, it is suggested that strengthening diabetes education and management are important strategies for treating T2DM patients in the Guangxi Zhuang population.

This study confirmed earlier findings by demonstrating that the risk of CVD increased with age in Zhuang T2DM patients [15]. The analysis of epidemiological data shows that the proportion of CVD patients among women, especially elderly women, has increased, but the standardized diagnosis and treatment and the improvement of prognosis are poor. Among diabetic patients, compared with men, the risk of coronary heart disease in women is 58% higher and the risk of death is also increased by 13% [16]. Studies have reported that lack of physical activity, smoking, and excessive drinking are the main risk factors for the sharp rise in cardiovascular and metabolic diseases [17]. Increasing physical activity significantly protects against the main risk factors for CVD and is also conducive to weight control [18]. A Chinese cohort study showed that maintaining moderate physical activity ≥150 minutes per week or high-intensity physical activity ≥75 minutes per week in adults can reduce the incidence of CVD [19]. A recent study in the UK showed that the risk of cardiovascular death was reduced by 29% when the physical activity level was gradually increased to a combination of moderate and high-intensity physical activity ≥150 minutes per week over five years [20]. Poor blood pressure control is a risk factor for CVD, consistent with previous findings [21], and continuous blood pressure compliance can reduce the risk of CVDs. Damage to the vascular endothelium produced by continuous high-pressure blood flow can be reduced, and atherosclerotic plaque formation can also be reduced when the blood pressure of hypertensive individuals is managed below 140/90 mmHg [22], thereby reducing the incidence of ischemic stroke [23] and acute myocardial infarction [24]. This shows that the treatment of hypertension should not only emphasize the initial targets but should also address continuous long-term blood pressure targets. Dyslipidemia is a significant risk factor for CVD. Several studies and guidelines have pointed out that LDL-C is a key pathogenic factor for atherosclerotic CVD, and dyslipidemia has been estimated to lead to an increase of 9.2 million cardiovascular events. In contrast, reducing blood lipid levels can significantly reduce the morbidity and mortality associated with CVD [25].

The present study showed that 58.94% of the patients had a moderate or high 10-year absolute risk of ICVD and 90.23% of the patients were treated with hypoglycemic drugs. Hypoglycemic drug therapy appears to be underused in terms of its potential benefits in reducing cardiovascular risk [26]. Diabetic patients are often complicated with hypertension, dyslipidemia, obesity, and other cardiovascular risk factors. Only improving blood sugar control cannot effectively delay the progress of diabetic macrovascular complications. Studies have shown that active intervention in CVD risk factors such as hypertension and dyslipidemia can reduce the risk of cardiovascular events in patients with T2DM. ACCORD researchers published the results of post-event epidemiological analysis of the research data [27], which showed that the increase of HbAlc was closely related to the increase of death risk. Every 1% increase of HbA1c on average would increase the all-cause mortality by 20%. The analysis results show that the mortality of people with HbA1c<7% is reduced among patients receiving intensive hypoglycemic therapy. After further analysis of the results of the ACCORD trial, the researchers mentioned that the increase in mortality only occurred in people whose blood sugar control was still unsatisfactory after intensive treatment, and most of these people were relatively old, with a long course of diabetes and high-risk groups with cardiovascular and cerebrovascular complications.

While ICVD is considered a significant predictor of 10-year cardiovascular risk in the overall Chinese population, it was not necessarily appropriate for the present study on the Zhuang population as this study is a cross-sectional survey rather than a 10-year study. Despite being a cross-sectional study, we found that the risk score was not related to the occurrence of CVD. This may suggest that the ICVD10-year absolute risk score does not apply to the Zhuang population. Therefore, further investigation is required into a suitable assessment scale for predicting cardiovascular risk in the Zhuang population.

There are limitations to this study. As the study subjects were not randomly sampled, there may be some selection bias. Diabetic retinopathy (DR), an important diabetic microangiopathy, was not included. Follow-up studies should be supplemented with DR screening. Furthermore, this study was cross-sectional. Future studies should address the integrated management of diabetes in hospitals and communities, and the metabolic management status of patients belonging to ethnic minorities in community hospitals should be addressed. These measures can promote patient self-management, increase comprehensive standards, and lessen the occurrence and progression of cardiovascular diseases while strengthening diabetes education for all patients.

In conclusion, the compliance levels of blood glucose, blood lipids, and blood pressure in the Zhuang population are abysmal, as is medication compliance. Hence, achieving comprehensive control of blood glucose, blood pressure, and blood lipids in Zhuang T2DM patients should be a direction for future research. The results of this study may serve as a warning to improve the clinical management of diabetic patients in the public health system in ethnic minority areas and thus also lower the economic burden of chronic diseases in China, given the observed increasing prevalence of T2DM.

## Figures and Tables

**Table 1:** Comparison of general data of patients [*n* (%), $\bar{\chi }$±*s*].

Group	Characteristics	Total *n*(%)	CVD (*n* = 161)	Non-CVD (*n* = 1814)	*t/F*	*P* value
Gender	Male	1328 (67.24)	122 (75.78)	1206 (66.48)	5.798	0.016
	Female	647 (32.76)	39 (24.22)	608 (33.52)		

	Age (y)	58.89 ± 11.24	61.66 ± 10.82	58.64 ± 11.25	−3.280	0.001
	SBP (mmHg)	139 ± 23	144 ± 24	138 ± 23	−3.392	<0.001
	DBP (mmHg)	79 ± 13	80 ± 13	79 ± 13	−1.004	0.316
	LDL-c (mmol/L)	3.14 ± 1.19	3.14 ± 1.05	3.12 ± 1.21	−0.176	0.861
	HDL-c (mmol/L)	1.24 ± 0.58	1.23 ± 0.40	1.24 ± 0.59	0.105	0.916
	TG (mmol/L)	2.31 ± 3.12	2.35 ± 2.86	2.31 ± 3.13	−0.167	0.867
	TC (mmol/L)	4.92 ± 3.95	4.75 ± 1.42	4.93 ± 4.10	0.558	0.577
	HbA1c (%)	8.85 ± 3.04	8.54 ± 2.84	8.88 ± 3.05	1.385	0.166
	Course of diabetes (year)	53.99 ± 11.38	55.35 ± 11.76	53.87 ± 11.35	−1.576	0.115
	Duration of diabetes (year)	6.01 ± 4.93	5.95 ± 5.03	6.01 ± 4.92	0.148	0.883

BMI (kg/m^2^)	≤23.9	972 (49.22)	78 (48.45)	894 (49.28)	0.431	0.806
	24–27.9	730 (36.96)	58 (36.02)	672 (37.05)		
	≥28	273 (13.82)	25 (15.53)	248 (13.67)		

10-year ICVD risk	Low risk	839 (42.48)	36 (22.36)	803 (44.26)	32.625	<0.001
	Moderate risk	468 (23.70)	53 (32.92)	415 (22.88)^a^		
	High risk	668 (33.82)	72 (44.72)	596 (32.86)^a^		

History of diabetes	No	1462 (74.03)	132 (81.99)	1330 (73.32)	5.780	0.015
	Yes	513 (25.97)	29 (18.01)	484 (26.68)		

Middle-intensity exercise	No	1599 (80.96)	111 (68.94)	1488 (82.03)	16.425	<0.001
	Yes	376 (19.04)	50 (31.06)	326 (17.97)		

Drinking	No	956 (48.41)	67 (41.61)	889 (49.01)	3.236	0.072
	Yes	1019 (51.59)	94 (58.39)	925 (50.99)		

Smoking	No	985 (49.87)	94 (58.39)	891 (49.12)	5.080	0.024
	Yes	990 (50.13)	67 (41.61)	923 (50.88)		

Glycemic control	HbA1c < 7	515 (26.08)	53 (32.92)	462 (25.47)	41.085	<0.001
	HbA1c ≥ 7 (but use hypoglycemic drugs)	1091 (55.24)	77 (47.83)	1014 (55.90)		
	HbA1c ≥ 7 (no hypoglycemic drugs)	369 (18.68)	31 (19.25)	338 (18.63)		

BP control	BP reached targets	904 (45.77)	35 (21.74)	869 (47.91)	41.085	<0.001
	BP not reached targets (but use antihypertensive drugs)	296 (14.99)	37 (22.98)	259 (2.28)^b^		
	BP not reached targets (no antihypertensive drugs)	775 (39.24)	89 (55.28)	686 (37.82)^b^		

Lipid control	LDL-c reached targets	604 (30.58)	16 (9.94)	588 (32.41)	39.195	<0.001
	LDL-c not reached targets (but use lipid-lowering drugs)	46 (2.33)	5 (3.11)	41 (2.26)^c^		
	LDL-c not reached targets (no lipid-lowering drugs)	1325 (67.09)	140 (86.96)	1185 (65.33)^c^		

Education (*n* (%))	≤Primary school	1102 (55.80)	101 (5.11)	1001 (55.18)	4.771	0.092
	Middle school	719 (36.41)	53 (32.92)	666 (36.71)		
	≥College	154 (7.80)	7 (4.35)	147 (8.10)		
Occupation (*n* (%))	Heavy labor	588 (29.77)	48 (29.81)	540 (29.77)	3.700	0.296
	Light labor	429 (21.72)	44 (27.33)	385 (21.22)		
	Mental labor	397 (20.10)	29 (18.01)	368 (20.29)		
	Others	561 (28.41)	40 (24.84)	521 (28.72)		

Monthly income/yuan (*n* (%))	≤5000	741 (37.52)	67 (41.61)	674 (37.16)	1.694	0.429
	5000–10000	654 (33.11)	53 (32.92)	601 (33.13)		
	≥10000	580 (29.37)	41 (25.47)	539 (29.71)		

*Note.* a: compared with the low-risk group, *P*  <  0.001; b: compared with the BP reached targets group, *P*  <  0.001; c: compared with the LDL-c reached targets group, *P*  <  0.01.

**Table 2:** Analysis of current drug treatment of patients with abnormal metabolic indicators (blood glucose, blood pressure, and blood lipid).

Group	Characteristics	CVD		Non-CVD	
		*n* (%)	Abnormality of blood glucose, blood pressure and blood lipid medication *n* (%)	*n* (%)	Abnormality of blood glucose, blood pressure and blood lipid medication *n* (%)
Abnormal blood glucose		138 (85.71)	107 (77.54)	1651 (90.01)	1312 (79.47)
	OHD only		37 (26.81)		485 (29.38)
	Insulin only		53 (38.41)		628 (38.04)
	OHD + insulin		17 (12.32)		199 (12.05)

Abnormal blood pressure		126 (78.26)	4 (3.17)	946 (52.15)	86 (4.74)
	ACEI/ARB		1 (0.08)		3 (0.32)
	Calcium channel		3 (2.38)		78 (8.25)
	*β*-blockers		0 (0.0)		5 (0.53)

Dyslipidemia		146 (90.68)	6 (4.11)	1244 (68.58)	59 (4.74)
	Statins		6 (4.11)		59 (4.74)

*Notes*. OHD: oral hypoglycemic drug; ACEI: ACE inhibitor; ARB: A II receptor antagonist.

**Table 3:** Binary logistic regression analysis of CVD influencing factors.

Group	Characteristics	*β*	Wald	OR (95% CI)	*P* value
	Age	0.033	15.272	1.033 (1.016, 1.050)	<0.001
	Gender	−0.912	16.785	0.402 (0.260, 0.621)	<0.001
	Middle-intensity exercise	−0.824	18.251	0.439 (0.300, 0.640)	<0.001
	Smoke	0.679	12.219	1.994 (1.361, 2.922)	<0.001

BP control	BP reached targets		28.140		<0.001
	BP not reached targets (but use antihypertensive drugs)	−1.055	24.954	0.348 (0.230, 0.527)	<0.001
	BP not reached targets (no antihypertensive drugs)	0.047	0.048	1.048 (0.686, 1.601)	0.827

Lipid control	LDL-c reached targets		26.746		<0.001
	LDL-c not reached targets (but use lipid-lowering drugs)	−1.413	26.590	0.244 (0.142, 0.417)	<0.001
	LDL-c not reached targets (no lipid-lowering drugs)	0.012	0.001	1.012 (0.383, 2.671)	0.981

## Data Availability

Data used to support this study are available on request.

## Additional Points

*Summary. *There are differences in dietary habits and diabetes-related gene phenotypes between Han and Zhuang nationalities in China. Zhuang people live in mountainous areas and like to grow tobacco and drink alcohol, and their blood sugar, blood pressure, and blood lipid are low, and their medication compliance is low.
